# Salivary Parameters Before and After Removable Complete Denture Rehabilitation: A Systematic Review and Meta‐Analysis

**DOI:** 10.1111/joor.70310

**Published:** 2026-09-20

**Authors:** Cátia Sufia Alves Freire de Andrade, Mariana Alves dos Santos, Mirtes Maria Ferreira Corrêa, Ayrton Geroncio Silva, Bruna Egumi Nagay, Valentim A. R. Barão, Raphael Cavalcante Costa, Guilherme Almeida Borges

**Affiliations:** ^1^ Department of Prosthodontics and Periodontology, Piracicaba Dental School Universidade Estadual de Campinas (UNICAMP) Piracicaba São Paulo Brazil; ^2^ Department of Prosthodontics and Periodontology, Bauru School of Dentistry University of São Paulo (USP) Bauru São Paulo Brazil; ^3^ Department of Prosthodontics, Saveetha Dental College and Hospitals, Saveetha Institute of Medical and Technical Sciences (SIMATS) Saveetha University Chennai Tamil Nadu India; ^4^ Department of Prosthodontics, School of Dentistry University of São Paulo (USP) São Paulo São Paulo Brazil

**Keywords:** dental prosthesis, dentures, pH, salivary flow

## Abstract

**Background:**

Salivary parameters are clinically relevant for complete denture (CD) lubrication, comfort, retention, and adaptation in edentulous patients. Although an immediate salivary response following CD insertion is biologically plausible, the magnitude and persistence of changes in salivary flow, pH, and biomarkers after treatment remain incompletely understood.

**Objective:**

To investigate whether the installation of new CDs influences salivary parameters (flow rate, pH and biomarkers) in edentulous patients, both under stimulated and unstimulated conditions.

**Methods:**

Five databases were searched to identify clinical studies comparing total salivary flow, salivary pH and biomarkers before and after CD installation. The ROBINS‐I tool was used to assess risk of bias, and Comprehensive Meta‐Analysis software (version 3.2) was used to perform the meta‐analysis; biomarker data were synthesized descriptively.

**Results:**

Twenty‐two studies were selected, comprising 645 edentulous patients rehabilitated with CDs, evaluated immediately after CD installation and at 2–3 months. Eight were included in the quantitative analysis. Six studies had a serious risk of bias, and 16 had a critical risk. The meta‐analyses showed significant increases in both stimulated and unstimulated salivary flow, as well as in salivary pH, immediately after CD insertion and after 2–3 months of use (*p* < 0.05). Descriptive analysis revealed time‐dependent changes in salivary biomarkers for inflammation, oxidative stress and immunity following CD installation.

**Conclusion:**

CD installation increased salivary flow, both stimulated and unstimulated, and salivary pH over time, with biological changes reflected by salivary biomarkers. However, because of the high risk of bias, these findings should be interpreted with caution.

## Graphical abstract

Graphical summary of the systematic review and meta‐analysis evaluating salivary parameters before and after the installation of complete dentures (CDs) in edentulous patients. The complete schematic design was created using BioRender.com under licence number UJ29I1R6RC.

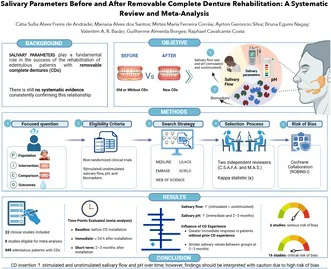

## 1 Introduction

Edentulism, the complete loss of natural teeth, represents a major global health burden associated with aging and socioeconomic disparities [1]. Prosthetic rehabilitation is therefore crucial for restoring oral function and quality of life [2]. Although removable complete dentures (CDs) are not the optimal treatment for complete edentulism, they remain widely used because they are affordable, accessible and able to restore essential oral functions [2, 3]. Successful rehabilitation with CDs depends on anatomical, physiological, and behavioural factors and on interactions between the CD and the biological environment [4].

Among biological factors, saliva plays a significant role in oral health and prosthetic function [5]. Composed of water, electrolytes, mucins, enzymes, immunoglobulins, and numerous proteins, saliva lubricates and protects oral tissues, contributes to taste and digestion, and buffers dietary and bacterial acids [6]. In CD wearers, a thin film of saliva between the intaglio CD base and the mucosa contributes to CD retention through a fluid seal, provides lubrication that reduces mucosal trauma, and helps maintain a balanced oral microbiome beneath the CDs [7, 8]. Consequently, changes in salivary secretion after CD installation may affect prosthesis biomechanics, patient comfort, mucosal health, including the risk of denture stomatitis, and even oral functions such as chewing and taste [9, 10].

Salivary flow comprises two clinically relevant states: unstimulated flow, which predominates during quiet wakefulness and reflects basal glandular activity, and stimulated flow, which increases in response to mastication, gustatory stimulation, or other sensory inputs and reflects the functional reserve of the salivary glands [11]. New CDs may influence both states through immediate sensory‐mechanical stimulation and subsequent physiological adaptation [12, 13]. Contact between the CD surface and the mucosa, tongue, and residual ridges can activate neural reflexes and increase salivary output, particularly during chewing or tasting [12]. Over time, adaptation to the prosthesis may alter glandular secretion patterns and salivary composition as functional demands and the oral environment change [13]. The magnitude and time course of these responses may also be influenced by prior CD experience and by patient‐level factors [5, 14]. Individuals without prior CD experience may exhibit acute increases in unstimulated and stimulated flow immediately after CD insertion [12], because the prosthesis represents a novel sensory stimulus [15]. In contrast, experienced wearers may exhibit smaller responses due to previous adaptation of the oral sensory and secretory systems [4, 5, 13, 16]. These differences are important for interpreting salivary responses and understanding patient adaptation, comfort and treatment success.

Several individual studies have investigated the effects of CD insertion on salivary outcomes, reporting changes in flow rate [4, 5, 8, 12, 13, 14, 15, 16, 17, 18, 19, 20, 21, 22], pH [5, 8, 9, 12, 13, 19, 23, 24] and biochemical composition [5, 9, 12, 17, 23, 25, 26, 27, 28, 29]. However, these studies were conducted independently, without comprehensive synthesis of the evidence. Although salivary changes after new CD installation are clinically recognized, the literature still lacks a systematic assessment of their consistency, magnitude and persistence. No systematic review has determined whether new CDs consistently modify salivary parameters compared with wearing old CDs or remaining without CDs. Therefore, this systematic review aimed to evaluate the available evidence on the impact of new CDs on salivary physiology in completely edentulous patients.

## 2 Materials and Methods

### 2.1 Protocol, Registration and Focused Question

The conduction of this systematic review followed the Preferred Reporting Items for Systematic Review and Meta‐Analysis (PRISMA) guidelines [30]. The focused question was formulated using the Population, Intervention, Comparison and Outcomes (PICO) framework. Thus, the PICO question was formulated as: ‘In completely edentulous patients rehabilitated with CDs (P), does the installation of new CDs (I), compared with wearing old CDs or having no CDs (C), result in changes in salivary parameters (O)?’ The primary outcomes included stimulated and unstimulated salivary flow and pH before and immediately after installation, as well as at 2–3 months post‐installation. Secondary outcomes included salivary biomarkers. The systematic review protocol was prospectively registered in the International Prospective Register of Systematic Reviews (PROSPERO) database [31] under the registration number CRD420250637192.

### 2.2 Eligibility Criteria

Based on the guiding question structure, eligible studies included non‐randomized clinical trials (N‐RCT) comprising between‐subjects and within‐subjects designs that evaluated changes in salivary parameters, including stimulated and unstimulated salivary flow, pH and biomarker parameters such as C‐reactive protein (CRP); tumour necrosis factor‐alpha (TNF‐α); nuclear factor kappa B (NF‐κB); myeloperoxidase (MPO); uric acid (UA); nitric oxide (NO); thiobarbituric acid reactive substances (TBARS); malondialdehyde (MDA); advanced oxidation protein products (AOPP); catalase (CAT); immunoglobulin E (IgE) and salivary IgA (IgA); salivary cortisol (SC) and salivary alpha‐amylase (SAA) in completely edentulous patients following the installation of new CDs. These studies were assessed to compare patients receiving new CDs with those wearing old CDs or none. Randomized controlled trials were not included because salivary outcomes are commonly assessed using a before–after design, in which patients serve as their own controls after CD installation. Furthermore, the exclusion criteria were applied: (I) in vitro, *in silico* and pre‐clinical studies in animal models; (II) literature reviews of any type, including systematic, narrative or integrative reviews; (III) meeting abstracts, case reports, case series; (IV) descriptive, observational and retrospective study designs; (V) participants who were not completely edentulous, such as those requiring removable partial dentures; (VI) studies involving patients with systemic diseases that could interfere with salivary parameters; and (VII) studies analysing saliva from specific salivary glands rather than whole saliva.

### 2.3 Information Sources and Search Strategy

The search was performed in five databases: MEDLINE (via PubMed), Embase, Web of Science, Latin American and Caribbean Health Sciences Literature (LILACS) and Scielo. Additional sources of articles were identified through clinical trial registries, manual searches, and Google Scholar and OpenGrey searches. Furthermore, no language or time restrictions were applied to the search. The search strategies (Table S1) for the PubMed database were formulated using MeSH terms, entry terms, and free‐text terms, combined through the Boolean operators “OR” and “AND”. Once finalized, the strategy was adapted to the syntax rules of the other databases. To ensure searches remained up to date, alerts were also created in each database to monitor the publication of potentially eligible articles.

### 2.4 Selection Process

Initially, the duplicate records were removed using Rayyan Software, developed by the Qatar Computing Research Institute (QCRI), and subsequently screened by two independent reviewers (C.S.A.F.A. and M.A.S.). Any disagreement was resolved through discussion with a third reviewer (G.A.B.). Studies with insufficient or unclear information were assessed in full text for final judgement. Additionally, potentially relevant titles/abstracts were also evaluated in full by the same reviewers (Figure 1). Based on the articles that met the inclusion criteria, information was extracted.

**FIGURE 1:**
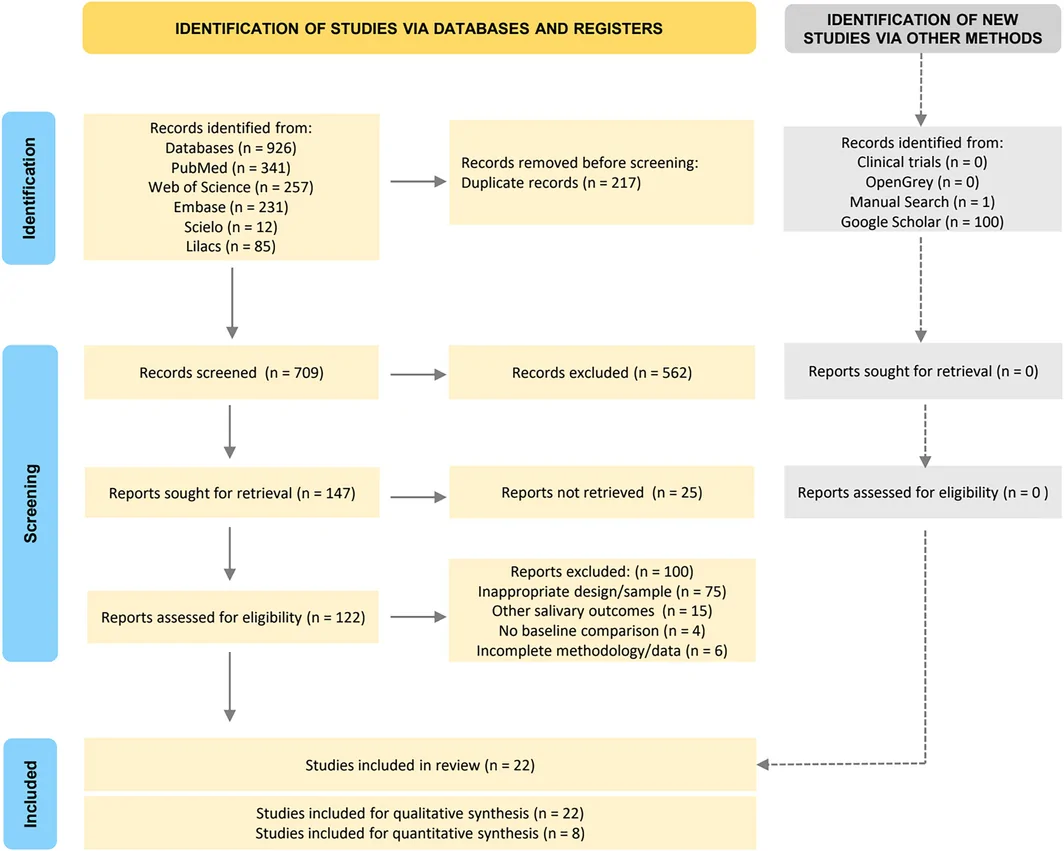
PRISMA flowchart of the studies' selection process.

### 2.5 Data Collection Process

Data were collected and organized using Microsoft Excel (2024) by two independent reviewers (C.S.A.F.A. and M.A.S.) to ensure accuracy. Any doubts were resolved through discussion with a third reviewer (G.A.B.). Data were organized into descriptive tables to group the dependent variables (unstimulated and stimulated salivary flow, pH and biomarkers). Some characteristics of the studies were also collected, including author/year, study design, sample size, age, gender, prior experience with CDs, systemic condition, follow‐up and saliva collection protocol. For data reported only in graphs, the web‐based tool WebPlotDigitizer was used to extract numerical data. The inter‐reviewer reliability during the selection process was measured using the kappa statistic (κ) with IBM SPSS Statistics software (v20.0; IBM Corp) to assess the consistency between the reviewers. In case of missing or unclear information, the authors were contacted up to three times via e‐mail.

### 2.6 Risk of Bias Assessment

Risk of bias was assessed by two reviewers (C.S.A.F.A. and M.A.S.). Thus, following the revised Cochrane Collaboration risk‐of‐bias tool, the Risk of Bias in Non‐randomized Studies—of Interventions (ROBINS‐I) tool was used to assess the risk of bias of the included studies [32]. The specific domains were divided into pre‐intervention, intervention and post‐intervention. In the pre‐intervention domain, confounders included systemic conditions known to affect salivary flow (e.g., diabetes, radiotherapy), neuromuscular disorders, lifestyle factors such as tobacco and alcohol use, medication intake and prior experience with CDs. For the intervention domain, we evaluated whether patient selection criteria were predefined, whether groups were comparable at baseline, whether sample size calculations were reported, and whether CD type, fabrication protocol and ridge characteristics were specified. We also verified whether follow‐up commenced uniformly across participants, with CD delivery defined as the starting point. Post‐intervention considerations included adherence to CD use, the duration of the adaptation period (≥ 2 months), monitoring and reporting of dropouts and reasons for withdrawal, and the balance of withdrawals across groups. Outcome assessment was evaluated based on the use of validated data collection methods, instrument calibration, standardized timing and assessor training. Selective reporting was assessed by checking the prespecified outcomes, the predefined statistical analyses and the acknowledgment of study limitations. The overall risk of bias was classified as low when these factors were adequately controlled, as serious when only partially addressed, and as critical when major confounders or methodological limitations remained. In cases of uncertainty, a third reviewer (G.A.B.) was consulted to achieve consensus.

### 2.7 Effect Measures and Synthesis Methods

For quantitative synthesis, the studies were grouped by methodological comparability and outcome similarity, yielding separate meta‐analyses for stimulated and unstimulated salivary flow and for stimulated and unstimulated salivary pH. Within each outcome, data were further stratified by time point (immediately after and 2–3 months after installation) and by patient experience with CDs (with or without prior experience). Continuous data (mean, standard deviation, total sample size and unit of measurement) were extracted for each subgroup and organized into structured tables, with each row corresponding to a study and each column representing one condition (before and after the installation) at a defined follow‐up period. Since all studies evaluated the same sample under two conditions, an intra‐participant correlation coefficient of 0.5 [33] was assumed using Comprehensive Meta‐Analysis v3.2 (Biostat, CMA). This value represents a realistic and conservative estimate for clinical pre‐post data and is consistent with the before‐after within‐subject design adopted by all included studies. Furthermore, the mean difference (MD) with 95% confidence interval (CI) was calculated using CMA. A random‐effects model was applied, and heterogeneity was assessed using the *I* ^2^ index [34].

### 2.8 Scatter Plot Analysis

Scatter plots were constructed to visually explore the distribution and temporal dynamics of salivary parameters (salivary flow and pH) across the included studies. For each eligible study, mean values and standard deviations of salivary flow (stimulated and unstimulated) and salivary pH were extracted and plotted at three predefined time points: baseline (before CDs installation), immediately after installation and at 2–3 months of follow‐up. The plots were generated using GraphPad Prism (version 8.0.2; GraphPad Software, San Diego, CA, USA), with mean values represented as data points and error bars indicating standard deviations.

## 3 Results

### 3.1 Study Selection

A total of 926 publications were initially identified (Figure 1). After removing 217 duplicates, 709 studies remained for title and abstract screening, of which 147 were selected for full‐text review. Inter‐reviewer reliability was assessed using κ, which indicated almost perfect agreement (κ = 0.85) [35]. Full‐text evaluation resulted in the exclusion of 100 studies for the following reasons: inappropriate study design or sample characteristics (*n* = 75), assessment of salivary outcomes not aligned with the review question (*n* = 15), absence of baseline data for comparison (*n* = 4), and incomplete methodology or missing essential data (*n* = 6). Ultimately, 22 studies were included in the synthesis of results [4, 5, 8, 9, 12, 13, 14, 15, 16, 17, 18, 19, 20, 21, 22, 23, 24, 25, 26, 27, 28, 29].

### 3.2 Characteristics of Studies

The characteristics of the included studies are presented in Table 1 and Table S2. Table 1 summarizes 17 of the 22 included studies that evaluated the primary outcomes. Table S2 presents the remaining included studies that assessed the secondary outcomes. Studies Bhat et al. (2014), Breseghelo et al. (2016) and Živković et al. (2020) are presented in both tables because they reported both primary and secondary outcomes [9, 17, 23]. The articles were published between 1997 and 2024. All 22 included studies were N‐RCTs [4, 5, 8, 9, 12, 13, 14, 15, 16, 17, 18, 19, 20, 21, 22, 23, 24, 25, 26, 27, 28, 29]. A total of 645 patients were evaluated, aged 28 to 80 years. Ten studies reported the mean age of participants, which ranged from 52.55 to 78.20 years [18, 23]. Regarding participant sex, ten studies did not specify this information [4, 8, 12, 14, 16, 19, 20, 24, 27, 28]; one included only men [25] and 11 involved both sexes [5, 9, 13, 15, 17, 18, 21, 22, 23, 26, 29]. Most studies reported that they included healthy volunteers [4, 5, 9, 12, 16, 17, 18, 20, 21, 25, 26, 27, 28], while nine studies did not describe participants' health status [8, 13, 14, 15, 19, 22, 23, 24, 29]. Concerning intervention characteristics, all studies rehabilitated participants with CDs in both arches. Among these, nine reported having patients with prior experience using CDs [5, 13, 18, 20, 21, 22, 24, 25, 29]; seven included patients without prior experience [4, 9, 12, 16, 19, 26, 28]; and six did not describe patients' prior CD experience [8, 14, 15, 17, 23, 27].

**TABLE 1:** Characteristics of the N‐RCTs included in the systematic review.

Authors, Years	Patients characteristics						Follow‐up					Saliva collection protocol
	Study design	Sample size (n)	Sex	Age (Range/Mean)	Previous experience	Systemic condition	Before	Hours after	Days after	Weeks after	Months after	
Bhat et al., 2014 [23]	N‐RCT	20	M (12) F (8)	52.55 ± 9.9	NR	NR	pH (U): 6.70 ± 0.25	NA	NA	NA	pH (U): 6.43 ± 0.19	Time: NR Methods: passive drooling Pre‐collection: No eating for 2 h before collection
Breseghelo et al. 2016 [17]	N‐RCT	19	M (5) F (14)	64.4 ± 8.3	NR	Healthy	S.F (U): 2.03 ± 0.44	S.F (U): 1.99 ± 0.48	NA	NA	S.F (U): 1.86 ± 0.49	Time: NR Method: A sterilized cylindrical cotton piece was placed in the sublingual region for 4 min Pre‐collection: NR
Devkota et al. 2021 [14]	N‐RCT	40	NR	60–70 50–60	NR	NR	S.F (U): 0.27 S.F (S): 0.59	S.F (U): 0.60 S.F (S): 0.93	S.F (U): 0.58 S.F (S): 0.86	S.F (U): 0.50 S.F (S): 0.75	NA	Time: 1:00–4:00 p.m. Methods: Spitting (U); chewing paraffin wax for 2 min (S) Pre‐collection: No eating, drinking, or smoking 1 h before collection
Ichigaya et al. 2023 [18]	N‐RCT	21	M (8) F (13)	78.2 ± 8.4	Yes	Healthy	S.F (U): 3.17 ± 2.87 S.F (S): 12.84 ± 7.26	NA	NA	NA	S.F (U): 4.88 ± 2.99 S.F (S): 14.07 ± 5.73	Time: 9:00–11:00 Methods: Spitting (U) and chewing gum (S) Pre‐collection: No eating or drinking (except water) within 2 h before sampling
Maheshwari et al. 2013 [19]	N‐RCT	50	NR	40–75	No	NR	S.F (U): 0.34 ± 0.02 S.F (S): 0.64 ± 0.03 pH (U): 7.377 ± 0.06 pH (S): 7.414 ± 0.05	S.F (U): 0.85 ± 0.80 S.F (S): 0.98 ± 0.07 pH (U): 7.498 ± 0.05 pH (S): 7.622 ± 0.06	NA	NA	S.F (U): 0.69 ± 0.02 S.F (S): 0.77 ± 0.02 pH (U): 7.427 ± 0.06 pH (S): 7.502 ± 0.04	Time: 08:00–10:00 a.m. Method: Chewing paraffin wax (S) Pre‐collection: No food or drink for 2 h; rinse mouth with 10 mL of distilled water for 5 s
Márton et al. 2004 [20]	N‐RCT	24	NR	67 ± 6.8	Yes	Healthy	S.F (U): 0.36 ± 0.33	NA	NA	S.F (U): 0.39 ± 0.35	NA	Time: NR Methods: Preweighed vessels (U), preweighed 8‐mm‐diameter filter paper discs (PS) Pre‐collection: Asked to refrain from eating and drinking for 2 h
Mary et al. 2020 [4]	N‐RCT	38	NR	50–70	No	Healthy	S.F (U): 2.8 ± 0.9	S.F (U): 1.6 ± 0.8	NA	NA	S.F (U): 2.9 ± 0.7	Time: NR Method: Spitting (U) Pre‐collection: Rinsing the mouth with water
Muddugangadhar et al. 2015 [12]	N‐RCT	50	NR	30–76	No	Healthy	S.F (U): 0.33 ± 0.01 S.F (S): 0.64 ± 0.03 pH (U): 7.37 ± 0.06 pH (S): 7.42 ± 0.05	S.F (U): 0.85 ± 0.03 S.F (S): 0.98 ± 0.07 pH (U): 7.50 ± 0.04 pH (S): 7.63 ± 0.04	NA	NA	S.F (U): 0.69 ± 0.02 S.F (S): 0.77 ± 0.02 pH (U): 7.42 ± 0.05 pH (S): 7.50 ± 0.04	Time: 9:00–11:00 a.m. Method: Paraffin wax chewing Pre‐collection: 2‐h fasting and mouth rinse with 10 mL of distilled water
Nikolopoulou et al. 2007 [24]	N‐RCT	60	NR	50–80	Yes	NR	pH (U): 6.89 ± 0.07	NA	NA	NA	pH (U): 7.14 ± 0.07	Time: 09:00–10:00 a.m. Method: refrain from swallowing(U) Pre‐collection: No food or drink or smoke before coming to the clinic.
Peltola et al. 1997 [21]	N‐RCT	42	M (11) F (31)	34–76	Yes	Healthy	S.F (U): 3.00 S.F (S): 8.00	NA	NA	NA	S.F (U): 3.00 S.F (S): 8.10	Time: NR Method: Spitting (U), Chewing paraffin (S) Pre‐collection: NR
Shekhar et al. 2018 [8]	N‐RCT	30	NR	40–70	NR	NR	S.F (U): 0.41 ± 0.10 pH (U): 6.93 ± 0.21	S.F (U): 0.73 ± 0.10 pH (U): 7.07 ± 0.21	NA	NA	NA	Time: 9:00–11:00 a.m. Method: Spitting method Pre‐collection: NR
Sonthalia et al. 2016 [15]	N‐RCT	21	M (6) F (15)	35–70 (M = 60)	NR	NR	S.F (U): 0.24 ± 0.16	S.F (U): 0.36 ± 0.16	NA	NA	S.F (U): 0.26 ± 0.13	Time: 09:00–11:00 a.m. Method: Spitting (U) Pre‐collection: No food or drink for 2 h; rinse mouth with 5 mL of distilled water for 5 s
Tango et al. 2018 [13]	N‐RCT	33	M (13) F (20)	64.4	Yes	NR	S.F (U): 2.1 S.F (S): 6.3 pH (U): 7.8 ± 0.44	NA	NA	S.F (U): 2.7 S.F (S): 8.2 pH (U): 8.02 ± 0.41	NA	Time: 09:00–11:00 a.m. Method: NR Pre‐collection: No CD worn during sleep; no eating, smoking, or cleaning CDs 2 h before collection
Wolff et al. 2004 [22]	N‐RCT	10	M (4) F (6)	45–80	Yes	NR	S.F (U): 0.48 ± 0.18 S.F (S): 0.81 ± 0.35	NA	S.F (U): 0.65 ± 0.21 S.F (S): 0.97 ± 0.34	S.F (U): 0.58 ± 0.19 S.F (S): 0.81 ± 0.29	NA	Time: 08:00–11:00 a.m. Methods: Expectorate (U) and chewing paraffin (S) Pre‐collection: No eating, drinking, smoking, or oral hygiene for 2 h before collection
Yurdukoru et al. 2001 [5]	N‐RCT	23	M (14) F (9)	44–76	Yes	Healthy	S.F (U): 0.26 ± 0.11 S.F (S): 0.79 ± 0.39 pH (U): 7.45 ± 0.54 pH (S): 7.65 ± 0.51	S.F (U): 1.10 ± 0.86 pH (U): 7.62 ± 0.48	NA	NA	S.F (U): 0.62 ± 0.50 S.F (S): 1.57 ± 0.95 pH (U): 7.73 ± 0.33 pH (S): 7.90 ± 0.34	Time: 08:00–10:00 a.m. Method: Chewing paraffin wax (S) Pre‐collection: No food or drink for 2 h; rinse mouth with 10 mL of distilled water for 5 s
Zaaba & Dhanraj, 2020 [16]	N‐RCT	15	NR	55–70	No	Healthy	S.F (U): 0.45 S.F (S): 0.83	S.F (U): 0.65 S.F (S): 0.97	NA	NA	NA	Time: NR Methods: Spitting (U), Chewing paraffin (S) Pre‐collection: Rinse mouth with 5 mL of distilled water for 10 s
Živković et al. 2020 [9]	N‐RCT	30	M (13) F (17)	65	No	Healthy	pH (U): 7.3 ± 0.5	pH (U): 7.7 ± 0.1	NA	NA	pH (U): 7.1 ± 0.1	Time: 1:00–2:00 pm Pre‐collection: Rinsing the mouth with water

*Note:* Stimulated and unstimulated salivary flow rates, expressed as mL/min.

Abbreviations: CDs, complete dentures; NR, not reported; S, stimulated; S.F, salivary flow expressed in mL/min; U, unstimulated.

The studies investigated changes in various salivary parameters following the placement of CDs, including unstimulated [4, 5, 8, 12, 13, 14, 15, 16, 17, 18, 19, 20, 21, 22, 24] and stimulated [5, 12, 13, 14, 16, 18, 19, 21, 22] salivary flow, salivary pH [5, 8, 9, 12, 23, 24] and biomarkers listed in Table S2. In the case of inflammatory biomarkers (CRP, TNF‐α, NF‐κB, and MPO) [23, 25, 27], most studies reported an increase at days, weeks and months after CD installation, as was also observed for oxidative stress biomarkers (UA, NO, TBARS, MDA, AOPP and CAT) [17, 23, 26, 27] and immunological markers (IgE and IgA) [25, 28]. However, in most studies evaluating stress and neuroendocrine response biomarkers (SC, SAA) [9, 29], a reduction was observed immediately and months after CD installation. The outcomes were assessed before and after CD insertion, with collection times ranging from immediately (≤ 24 h), days (< 7 days), weeks (< 4 weeks) and months (< 12 months) following installation.

The most used methods to evaluate salivary flow were the traditional spitting method for unstimulated saliva [4, 8, 9, 14, 15, 16, 18, 21] and paraffin chewing for stimulated saliva [5, 12, 14, 16, 19, 21, 22]. Most studies described adherence to pre‐collection protocols, such as fasting from food and beverages for at least 30 min, rinsing with distilled water, and collecting samples between 8:00 and 11:00 a.m., to minimize variation in salivary concentration due to circadian rhythms [4, 5, 12, 13, 14, 15, 16, 17, 18, 19, 20, 22, 23, 24, 25, 26, 27, 28, 29]. The studies reported increases in both stimulated and unstimulated salivary flow and pH, as well as significant changes in key salivary biomarkers following CD placement.

### 3.3 Risk of Bias of Included Studies

The risk of bias of the included studies was assessed using the ROBINS‐I tool summarized in Figure 2. Six trials were classified as having a serious risk of bias [13, 19, 23, 24, 25, 27] and 16 trials were classified as having a critical risk of bias [4, 5, 8, 9, 12, 14, 15, 16, 17, 18, 20, 21, 22, 26, 28, 29]. The main methodological limitations were related to: (i) confounding bias, due to the lack of control over at least one important domain; (ii) intervention classification bias, resulting from insufficient detail and clarity in describing the intervention performed; and (iii) measurement bias, as outcomes were assessed by examiners aware of the interventions received by the participants. Due to the high risk of bias and heterogeneity among the included studies, a formal GRADE assessment was not undertaken.

**FIGURE 2:**
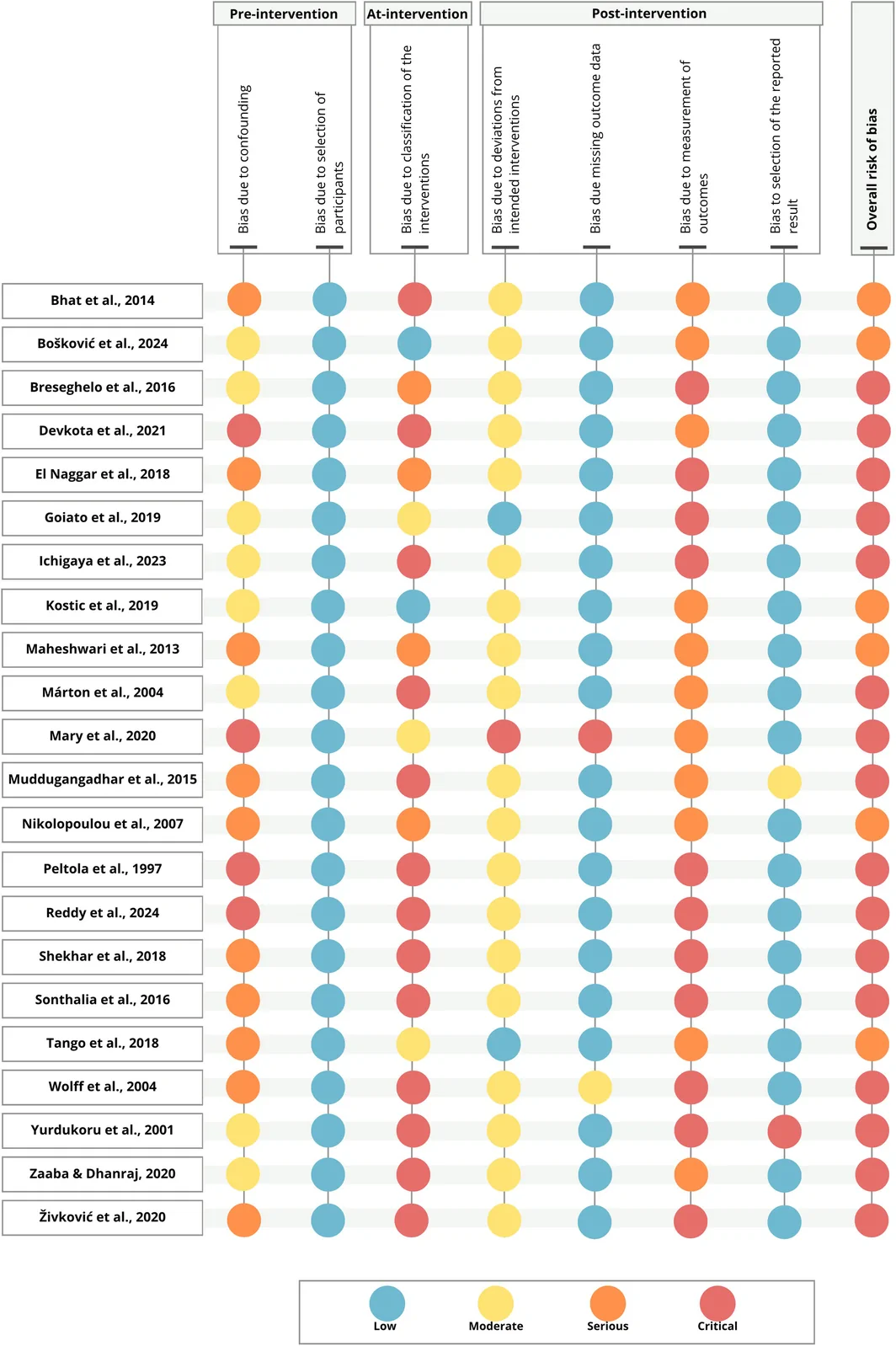
Risk of bias of the studies included in the systematic review.

### 3.4 Meta‐Analysis

#### 3.4.1 Unstimulated and Stimulated Salivary Flow

Of the 22 included studies, eight were selected for quantitative synthesis and grouped based on similar data and measurements [5, 8, 9, 12, 15, 17, 18, 19]. These were stratified according to patients' prior experience with CDs, separating experienced and inexperienced users. To ensure comparability, only studies that used standardized collection methods were included: unstimulated saliva was generally collected by spitting, whereas stimulated saliva was obtained by chewing paraffin wax or chewing gum. Only studies reporting salivary flow in mL/min and salivary pH measured with a digital pH meter were considered for the synthesis. Studies that reported only biomarkers as outcomes were excluded from this analysis, as they assessed distinct, non‐comparable outcomes [25, 26, 27, 28, 29]. This was primarily due to heterogeneity in analytical methods (ELISA, spectrophotometry, colorimetric assays, etc.), units of measurement (nm/mL, μM, mg/dL, U/ml, μmol/mg, etc.), and follow‐up time points, which precluded reliable quantitative synthesis (Table S2). In addition, studies presenting incomplete numerical data or assessing outcomes at non‐comparable follow‐up time points [20, 22, 23, 24] were also excluded.

According to the meta‐analysis data, unstimulated salivary flow, assessed before and immediately after the placement of CDs, showed a statistically significant increase in individuals without prior experience (MD = 0.443; 95% CI = 0.277–0.609; *p* < 0.001; *I* ^2^ = 98%; Figure 3A). A significant increase was also observed when all patients were analysed, regardless of prior CD experience, with a slightly larger effect size (MD = 0.496; 95% CI = 0.340–0.652; *p* < 0.001; *I* ^2^ = 97%). However, analysis of individual changes demonstrated a more pronounced effect among experienced CD wearers (MD = 0.840; 95% CI = 0.509–1.171; *p* < 0.001; Figure 3B). Two to 3 months after CD placement, the increase in unstimulated salivary flow was maintained, with the effect present in experienced patients (MD = 0.167; 95% CI = 0.077–0.257; *p* < 0.001; *I* ^2^ = 82%), inexperienced patients (MD = 0.293; 95% CI = 0.257–0.330; *p* < 0.001; *I* ^2^ = 98%; Figure 3C), and pooled patients (MD = 0.237; 95% CI = 0.113–0.361; *p* < 0.001; *I* ^2^ = 87%; Figure 3C).

**FIGURE 3:**
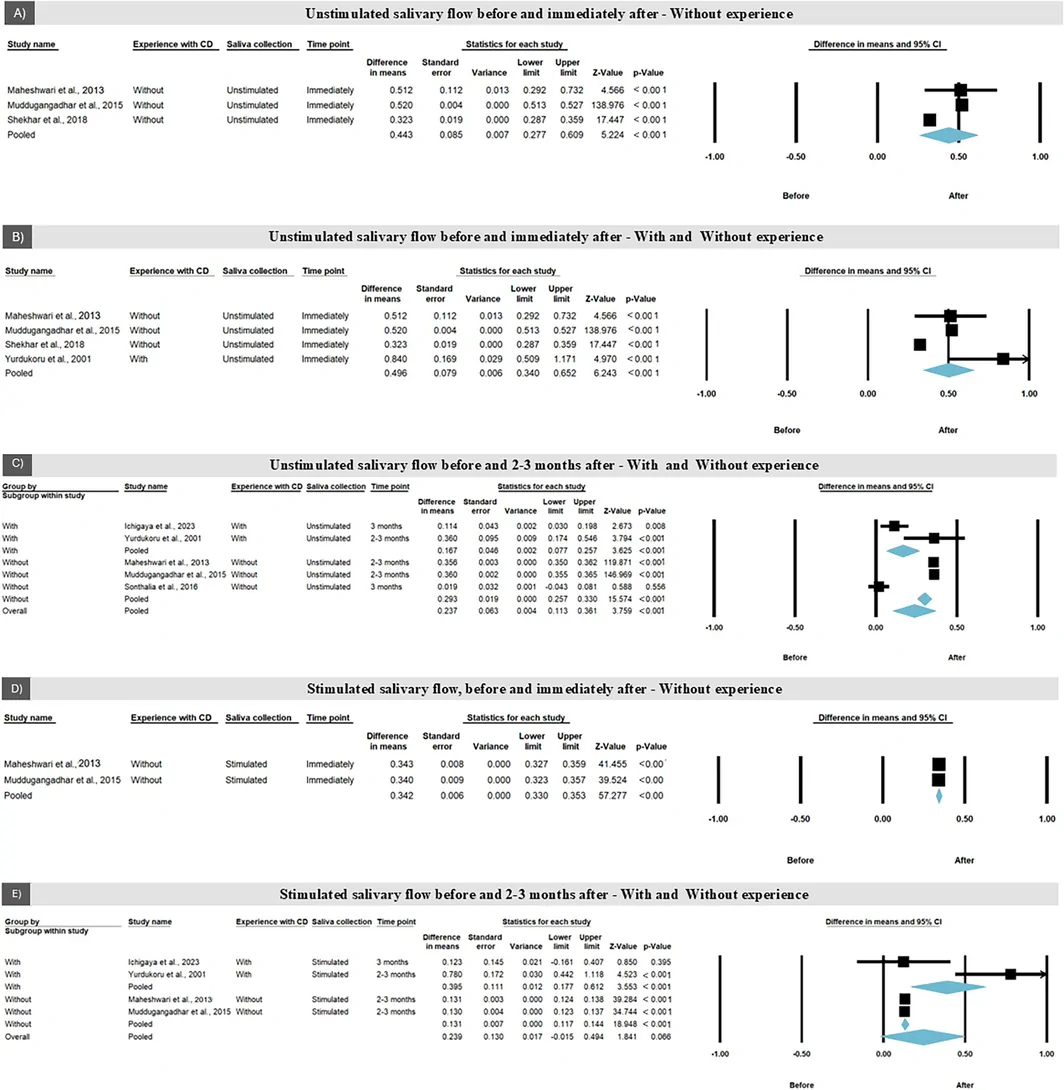
Random‐effects meta‐analyses of salivary flow before and after CDs installation. (A–C) Unstimulated flow: (A) immediately after in patients without prior experience; (B) immediately after in patients with/without prior experience; (C) 2–3 months after in patients with/without prior experience. (D–E) Stimulated flow: (D) immediately after in patients without prior experience; (E) 2–3 months after in patients with/without prior experience.

Stimulated salivary flow, assessed before and immediately after the placement of CDs, showed a significant increase in individuals without prior CD experience (MD = 0.342; 95% CI = 0.33–0.353; *p* < 0.001; *I* ^2^ = 0; Figure 3D). When stimulated salivary flow measured 2–3 months after CD installation was compared with baseline values, significant increases were observed in both experienced (MD = 0.395; 95% CI = 0.177–0.612; *p* < 0.001; *I* ^2^ = 88%) and inexperienced individuals (MD = 0.131; 95% CI = 0.117–0.144; *p* < 0.001; *I* ^2^ = 0; Figure 3E). Conversely, no significant differences in stimulated salivary flow were observed between participants with and without prior experience at the 2–3‐month follow‐up (MD = 0.239; 95% CI = −0.015–0.494; *p* = 0.066; *I* ^2^ = 78%; Figure 3E).

When unstimulated and stimulated salivary flow were compared, regardless of prior CD experience, a statistically significant difference was observed immediately after CD installation (MD = 0.340; 95% CI = 0.315–0.365; *p* < 0.001; *I* ^2^ = 69%; Figure S1A). A significant difference was also detected at 2–3 months after CD installation when compared with baseline values measured before installation (MD = 0.122; 95% CI = 0.1–0.144; *p* < 0.001; *I* ^2^ = 85%; Figure S1B).

#### 3.4.2 Unstimulated and Stimulated Salivary pH

Unstimulated salivary pH showed a statistically significant increase in patients without prior CD experience immediately after the placement of CDs, compared to baseline values (MD = 0.135; 95% CI = 0.104–0.166; *p* < 0.001; *I* ^2^ = 65%; Figure 4A). Including patients with prior experience maintained the significant increase, albeit with a slightly smaller effect (MD = 0.134; 95% CI = 0.105–0.164; *p* < 0.001; *I* ^2^ = 57%; Figure 4B). When comparing baseline values to those obtained 2–3 months after CD placement in patients without prior experience, the increase in unstimulated salivary pH was statistically significant (MD = 0.041; 95% CI = 0.010–0.073; *p* = 0.009; *I* ^2^ = 77%; Figure 4C). Similarly, when patients with prior experience were included, the pooled increase remained statistically significant (MD = 0.046; 95% CI = 0.008–0.084; *p* = 0.017; *I* ^2^ = 79%; Figure 4D) compared with baseline.

**FIGURE 4:**
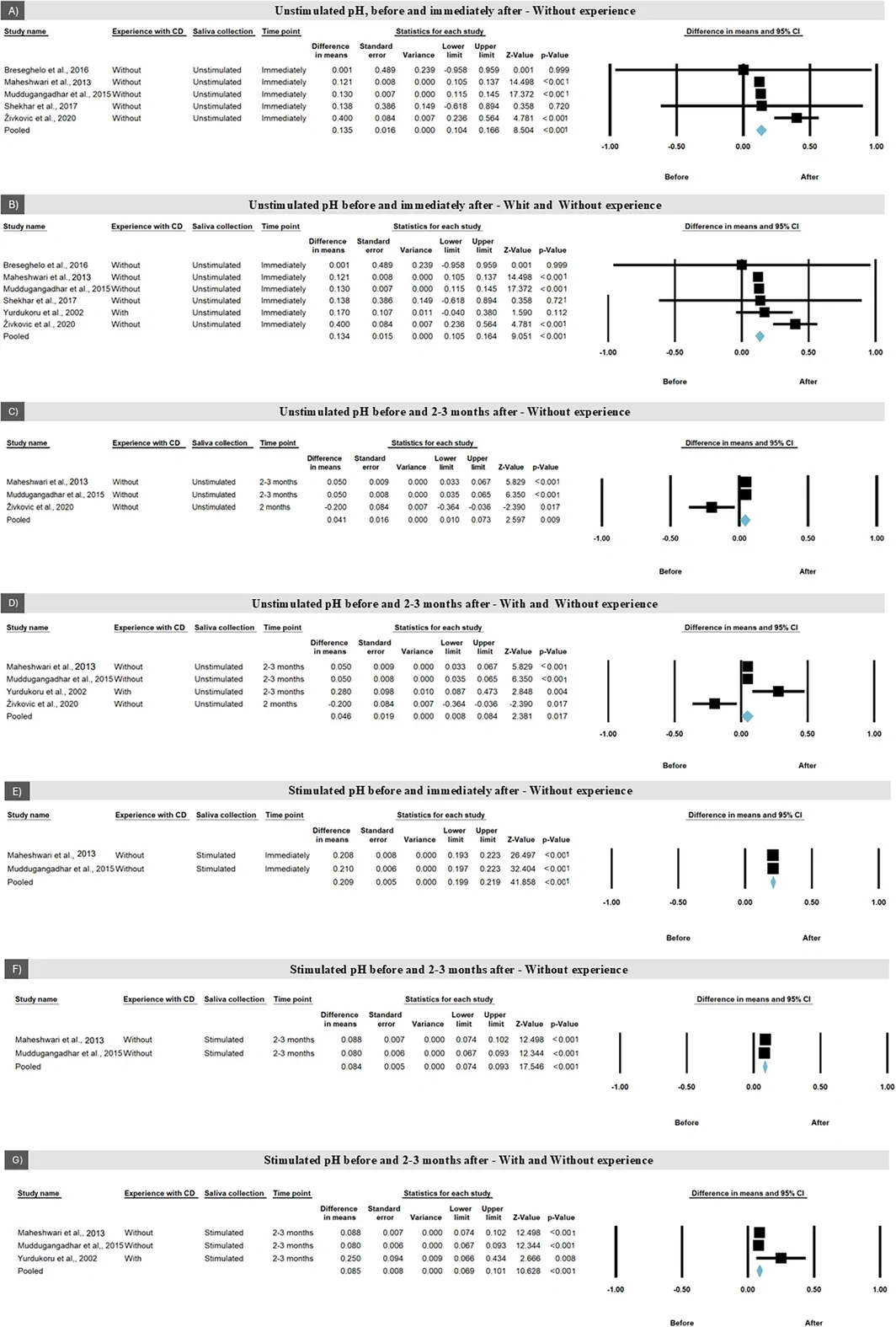
Random‐effects meta‐analyses of salivary pH before and after CDs installation. (A–D) Unstimulated pH: (A) immediately after in patients without prior experience; (B) immediately after in patients with/without prior experience; (C) 2–3 months after in patients without prior experience; (D) 2–3 months after in patients with/without prior experience. (E–G) Stimulated pH: (E) immediately after in patients without prior experience; (F) 2–3 months after in patients without prior experience; (G) 2–3 months after in patients with/without prior experience.

Similar to what was observed for unstimulated salivary pH, stimulated salivary pH also increased significantly in patients without prior CD experience immediately after the placement of CDs, compared to baseline values (MD = 0.209; 95% CI = 0.199–0.219; *p* < 0.001; *I* ^2^ = 0%; Figure 4E). This pattern persisted when comparing baseline values to those obtained 2–3 months after CD placement (MD = 0.084; 95% CI = 0.074–0.093; *p* < 0.001; *I* ^2^ = 0%; Figure 4F).

The data also indicated a significant difference in stimulated salivary pH between participants with and without prior CD experience at 2–3 months after CD installation (MD = 0.085; 95% CI = 0.069–0.101; *p* < 0.001; *I* ^2^ = 48%; Figure 4G). Statistically significant differences between unstimulated and stimulated salivary pH were observed immediately after CD installation. This was the case both when only patients without previous CD experience were pooled (MD = 0.274; 95% CI = 0.113–0.435; *p* < 0.001; *I* ^2^ = 26%; Figure S2A) and when patients with and without previous experience were analysed together (MD = 0.234; 95% CI = 0.153–0.314; *p* < 0.001; *I* ^2^ = 10%; Figure S2B). The same trend was observed 2 to 3 months after installation, again for patients without previous CD experience (MD = 0.05; 95% CI = 0.003–0.097; *p* = 0.031; *I* ^2^ = 91%; Figure S2C) and for the combined pool of patients with and without such experience (MD = 0.06; 95% CI = 0.013–0.108; *p* = 0.013; *I* ^2^ = 89%; Figure S2D).

### 3.5 Scatter Plot

In addition to the quantitative analysis presented in the forest plots, which summarize the combined effect sizes for the outcomes across the included studies, scatter plots were created to visually explore associations among time points, salivary stimulation types and prior experience with CDs. Figure 5 presents a detailed analysis of salivary flow and pH, both stimulated and unstimulated, assessed before, immediately after and 2–3 months following CD installation. Across studies, salivary flow levels were comparable, although isolated variations were observed, which may be attributable to methodological differences and sample size. In the first follow‐up period (immediately after CD installation), both types of salivary flow increased; however, at the 2‐ to 3‐month intervals, unstimulated salivary flow showed a slight reduction compared to the first period, while remaining above baseline values (before placement). In contrast, stimulated salivary flow increased continuously over time. Regarding stimulated and unstimulated salivary pH, the studies showed comparable levels at each time point, except for the study by Breseghelo et al. [17], in which unstimulated salivary pH values were substantially lower than those observed in the other studies. Nevertheless, in the remaining studies, a slight increase in both types of salivary pH was observed over the follow‐up period.

**FIGURE 5:**
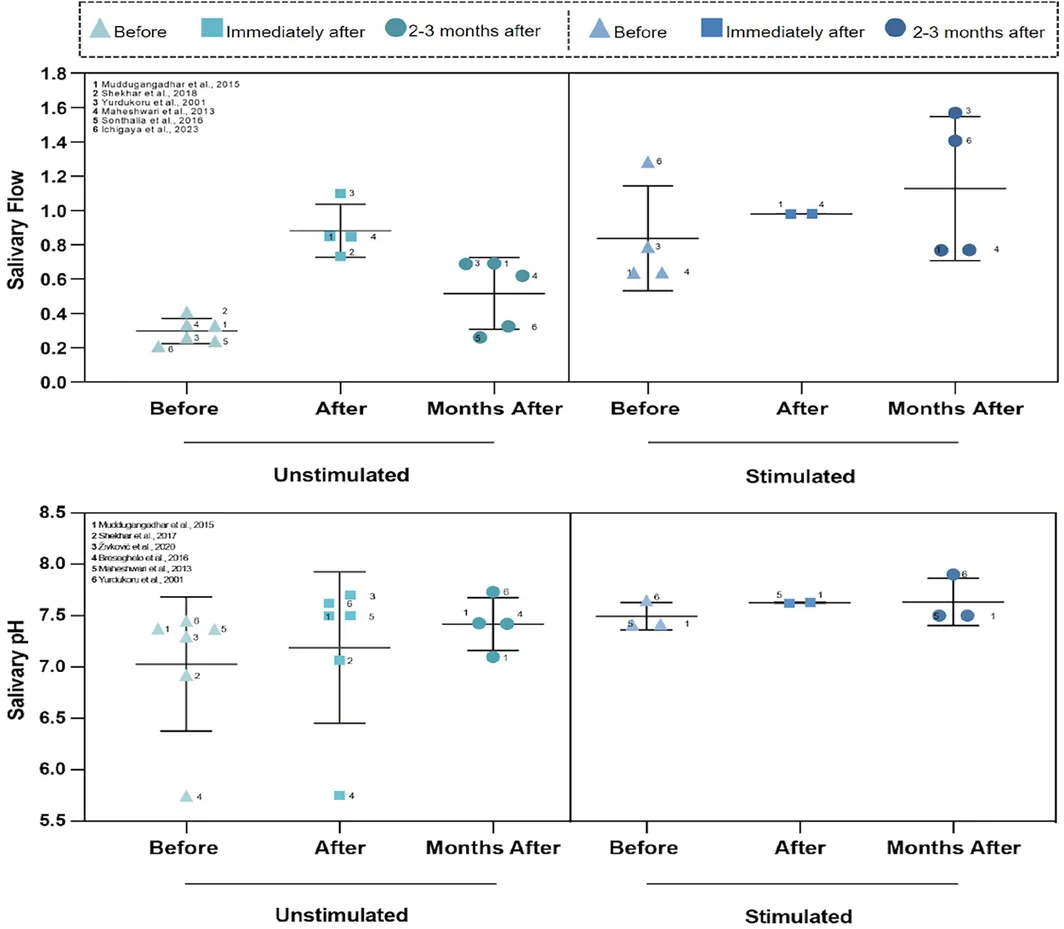
Scatter plots illustrate the mean and standard deviation of salivary flow and pH, both stimulated and unstimulated, before, immediately after and 2–3 months after CD installation in the included studies.

## 4 Discussion

Saliva plays a critical functional role in CD retention, producing cohesion, adhesion, lubrication, and surface tension, thereby providing a physical basis for stabilizing the CDs on the mucosal surface [7]. These effects interact with CD design, muscular control, patient adaptation and psychological acceptance [36]. Thus, saliva integrates these factors and supports effective adaptation and long‐term CD use [11]. At CD placement, saliva film mediates the initial interaction between the CD base and the supporting mucosa, contributing to the peripheral seal, retention and patient comfort [5, 12]. Therefore, understanding the distinct roles of unstimulated and stimulated saliva is essential for evaluating CD rehabilitation. A recent systematic review reported a reference salivary flow rate of approximately 0.82 mL/min for unstimulated flow and 1.48 mL/min for total stimulated flow in healthy adults [37]. In our analysis, edentulous patients showed lower baseline values, averaging 0.38 mL/min for unstimulated flow and 0.95 mL/min for stimulated flow, both below those reported for dentate patients [38]. This reduction may result from impaired mastication, which reduces mechanical and gustatory stimulation, the loss of periodontal input, and salivary gland hypofunction associated with chronic disuse [11, 39]. Limited adaptation to new CDs in studies with short follow‐up periods may also have influenced salivary flow measurements. Overall, these findings demonstrate the physiological impact of edentulism and address the limited availability of reference data on salivary parameters in this population [37].

Although the values remained below the physiological reference levels for dentate individuals, installing new CDs resulted in significant improvements in both salivary flow and pH. In the unstimulated flow, a clear, statistically significant increase was observed immediately after the CD was inserted. This pattern suggests that the CDs behave as a foreign body, activating oral mechanoreceptors and eliciting reflex salivation [15]. Interestingly, this effect was more pronounced in patients with prior denture experience than in those who were edentulous for the first time. Repeated exposure to a denture can condition the neural circuits that control salivation, making the reflex faster and stronger when the stimulus is reintroduced. Prior and continuous stimulation also helps preserve glandular secretory capacity [40], whereas prolonged periods without denture stimulation can lead to partial atrophy due to disuse [41]. Thus, CDs may support not only speech, swallowing, and mastication but also salivary function and stomatognathic adaptation.

The increases in salivary flow persisted for 2–3 months, although the magnitude is slightly lower than in the immediate period, suggesting stabilization at a new functional baseline. This adaptation likely reflects a balance between glandular reactivation and structural limitations associated with long‐standing edentulism, such as acinar atrophy and reduced glandular reserve capacity [42]. Previous studies indicate that approximately 3 months may be required for patients to achieve functional balance with new CDs, which may explain the stabilization observed in our meta‐analysis [12, 43]. Despite these increases, salivary flow did not reach dentate levels. This may be related to the reduced masticatory function commonly observed in edentulous patients rehabilitated with removable CDs [4]. Because mechanical and gustatory stimulation are major triggers for reflex salivation, impaired chewing efficiency may limit salivary secretion and reduce taste perception, contributing to lower flow rates [11].

The effect on stimulated salivary flow was even more pronounced and consistent than that observed for unstimulated flow. Individuals without prior CD experience demonstrated a marked increase in salivary secretion, reflecting the strong influence of mastication, particularly on parotid gland activity. At 2–3 months after CD installation, these improvements were maintained in both experienced and inexperienced patients, with no significant differences between groups. This pattern is consistent with a functional adaptation to the new CDs, in which progressive recovery of masticatory efficiency leads to stabilization of salivary stimulation [4, 12]. Our meta‐analysis further supports this interpretation, indicating that standardized masticatory stimulation tends to normalize glandular responses over time, regardless of prior CD experience. Clinically, this functional adaptation is relevant, as stimulated saliva plays a central role in CD lubrication, comfort, taste perception and overall oral function [11, 18]. However, these implications should be interpreted cautiously, since the included studies did not directly evaluate prosthetic performance or patient‐reported outcomes.

Stimulated secretion showed greater increases than unstimulated secretion both immediately and at 2–3 months. This pattern is physiologically coherent because stimulated flow is primarily mediated by the parotid glands and driven by masticatory activity, which activates mechanoreceptors and reflex pathways [12]. CD insertion restores functional chewing stimulation, re‐engaging these pathways. In contrast, unstimulated flow depends more on basal autonomic tone and residual glandular capacity, both of which are more susceptible to long‐term disuse and structural changes associated with edentulism.

The observed increases in salivary pH are closely related to the changes in salivary flow documented in our analysis. In healthy dentate individuals, salivary pH typically ranges from approximately 6.2 to 7.6, with oral pH maintained near neutrality (6.7–7.3) by efficient salivary buffering mechanisms [38]. In the present analysis, the baseline pH values in edentulous patients before CD insertion were already within this physiological range, clustering around the lower neutral values. After CD placement, mean salivary pH increased consistently, both immediately after insertion and at the 2–3‐month follow‐up, indicating a shift toward higher neutral pH rather than a transition from an acidic to a normal condition. This increase paralleled the rise in salivary flow observed after CD insertion, as higher secretion rates favour greater bicarbonate availability and dilution of acidic components [24]. This effect was more evident under stimulated conditions. Clinically, this modest increase may support a more stable oral environment and support mucosal health, potentially reducing the risk of CD‐associated opportunistic infections, such as *Candida* spp. [44, 45].

The scatter plots complemented the meta‐analyses by illustrating the distribution and temporal patterns of salivary flow and pH across studies. Although the overall trends were broadly consistent with the pooled estimates, substantial between‐study variability was evident. These plots therefore support the interpretation of the meta‐analytic findings while highlighting the potential influence of methodological and clinical heterogeneity.

Beyond salivary flow and pH, biomarker analysis suggests additional biological responses during the adaptation period to CD [9, 25, 26, 27, 28, 29]. Inflammatory biomarkers showed a non‐uniform pattern, with TNF‐α increasing from day 3 onward and NF‐κB increasing across all reported follow‐up periods, whereas MPO fluctuated over time [25, 27]. A similar trend was observed for oxidative stress‐related biomarkers, since TBARS and MDA increased after CD insertion, AOPP showed an initial decrease followed by subsequent increases, and catalase activity remained below baseline throughout follow‐up in one study [26, 27]. Immunological findings were also variable, with salivary IgA increasing over time, whereas IgE remained unchanged at all evaluated time points [25, 28]. Stress‐related biomarkers likewise behaved inconsistently across studies, with salivary cortisol decreasing one month after CD insertion, whereas salivary alpha‐amylase decreased in one study but increased in another [9, 26, 29]. Taken together, these findings suggest that adaptation to CDs involves measurable biological responses beyond mechanical adjustment, although the direction and magnitude of these changes were not fully consistent across studies.

From a clinical standpoint, CD rehabilitation may influence salivary physiology. The observed increases in salivary flow and pH appear biologically favourable and may improve lubrication and oral comfort during CD adaptation. However, because the included studies did not consistently assess CD retention, masticatory performance, speech, patient‐reported comfort or quality of life, the magnitude of the clinical benefit cannot be established. These findings should be interpreted cautiously because of substantial heterogeneity, particularly for unstimulated flow (*I* ^2^ > 80%). This variability may reflect differences in saliva collection protocols, patient age, CD adaptation, material properties and functional performance. Most studies also focused on short‐ to intermediate‐term follow‐up, while data from the immediate post‐installation period remain limited, despite frequent reports of increased salivary flow during this phase. In contrast, the absence of heterogeneity in the immediate stimulated flow analysis suggests that mastication‐driven secretion is a reproducible response that is relatively insensitive to methodological variation.

Most studies presented a serious or critical risk of bias because of inadequate control of confounding factors, incomplete participant characterization, insufficient intervention standardization, and limited reporting of outcome assessment and follow‐up. Systemic conditions, medication use, smoking, alcohol consumption, neuromuscular disorders, and previous CD experience may have influenced baseline salivary flow and pH and the magnitude of changes after CD insertion. Consequently, the observed effects may have been overestimated or underestimated. These factors may also have contributed to the variability in salivary biomarkers. Limited blinding and outcome standardization may have further affected the measurements. Therefore, some changes may not be attributable exclusively to CD insertion, although the overall pattern remained biologically plausible and relatively consistent. The evidence was also limited by the small sample size: 22 studies included only 645 participants, and eight were eligible for quantitative synthesis. This reduces the precision and external validity of the pooled estimates and increases the possibility of small‐study effects. Future studies should use standardized protocols and evaluate both immediate and longitudinal responses to refine effect estimates and optimize prosthetic strategies for preserving and restoring salivary function.

## 5 Conclusion

In completely edentulous patients, the insertion of new complete dentures suggests significant improvements in salivary parameters compared with wearing old dentures or no dentures. Both unstimulated and stimulated salivary flow increased immediately after denture insertion, with stimulated flow exhibiting a greater magnitude and more sustained improvement. Salivary pH also increased. Despite this partial restoration of salivary flow and buffering capacity, salivary parameters remained below the levels typically observed in dentate individuals.

## Author Contributions

**Cátia Sufia Alves Freire de Andrade:** conceptualization, methodology, data curation, investigation, writing – original draft. **Mariana Alves dos Santos:** conceptualization, methodology, data curation, investigation, writing – original draft. **Mirtes Maria Ferreira Corrêa:** conceptualization, methodology, data curation. **Ayrton Geroncio Silva:** conceptualization, methodology, data curation. **Bruna Egumi Nagay:** supervision, writing – review and editing. **Valentim A. R. Barão:** supervision, writing – review and editing. **Raphael Cavalcante Costa:** supervision, writing – review and editing. **Guilherme Almeida Borges:** supervision, writing – review and editing, formal analysis.

## Funding

This work was supported by Conselho Nacional de Desenvolvimento Científico e Tecnológico (444361/2024‐3) and Universidade Estadual de Campinas (01/2023).

## Conflicts of Interest

The authors declare no conflicts of interest.

## Supporting information


**Figure S1:** Forest plots showing differences in unstimulated and stimulated salivary flow before and after complete denture (CD) insertion.
**Figure S2:** Forest plots of the mean difference in unstimulated and stimulated salivary pH before and after complete denture (CD) insertion.
**Table S1:** Search strategy applied in each database.
**Table S2:** Salivary biomarkers reported in clinical studies, with mean and standard deviation at different time points. Follow‐up is shown in hours (h), days, weeks (w), and months (m) after intervention. “NA” indicates data not available.

## Data Availability

The datasets generated and analysed during the current study are available from the corresponding author upon reasonable request.
