# Supplementary material for: Salivary Parameters Before and After Removable Complete Denture Rehabilitation: A Systematic Review and Meta‐Analysis
Source: J Oral Rehabil. 2026 Sep 20;53(12):2474–90. doi: 10.1111/joor.70310 (PMC13755657; doi:10.1111/joor.70310)
Supplement: Supplementary file 1 [file JOOR-53-2474-s001.docx]

**Supplementary data**

**Salivary Parameters Before and After Removable Complete Denture Rehabilitation: A Systematic Review and Meta-Analysis**

Saliva and pH in Denture Patients

Cátia Sufia Alves Freire de Andrade^1^; Mariana Alves dos Santos^1^; Mirtes Maria Ferreira Corrêa^1^; Ayrton Geroncio Silva^1^; Bruna Egumi Nagay^2^; Valentim A. R. Barão^1,3^; Raphael Cavalcante Costa^4^; Guilherme Almeida Borges^4^

^1^ Department of Prosthodontics and Periodontology, Piracicaba Dental School, Universidade Estadual de Campinas (UNICAMP), Piracicaba, São Paulo, Brazil.

^2^ Department of Prosthodontics and Periodontology, Bauru School of Dentistry, University of São Paulo (USP), Bauru, São Paulo, Brazil.

^3^ Department of Prosthodontics, Saveetha Dental College and Hospitals, Saveetha Institute of Medical and Technical Sciences (SIMATS), Saveetha University, Chennai, Tamil Nadu 600077, India.

^4^ Department of Prosthodontics, School of Dentistry, University of São Paulo (USP), São Paulo, São Paulo, Brazil.

**Table S1**. Search strategy applied in each database.

| **Database** | **Search Strategy** |
| --- | --- |
| Pubmed  (n=341) | #1: ("Saliva"[Title/Abstract] OR "salivas"[Title/Abstract] OR "stimulated whole saliva"[Title/Abstract] OR "salivary flow "[Title/Abstract] OR "saliva flow"[Title/Abstract] OR "salivary flow rate"[Title/Abstract] OR "resting whole saliva"[Title/Abstract] OR "saliva flow rate"[Title/Abstract] OR "unstimulated flow rates"[Title/Abstract] OR "unstimulated salivary flow"[Title/Abstract] OR "salivary"[Title/Abstract] OR "salivary glands"[Title/Abstract] OR "gland, salivary"[Title/Abstract] OR "glands, salivary"[Title/Abstract] OR "salivary gland"[Title/Abstract] OR "Ph"[Title/Abstract] OR "Hyposalivation"[Title/Abstract]) OR (saliva[MeSH Terms])) OR (salivary glands[MeSH Terms])  #2: (denture, complete[MeSH Terms]) OR (Denture, Complete, Lower[MeSH Terms])) OR (Denture, Complete, Upper[MeSH Terms])) OR ("Denture, Complete"[Title/Abstract] OR "Complete Denture"[Title/Abstract] OR "Complete Dentures"[Title/Abstract] OR "Dentures, Complete"[Title/Abstract] OR "Denture, Complete, Lower"[Title/Abstract] OR "Denture, Complete, Upper"[Title/Abstract]) |
| Web of Science  (n=257) | #1: TS=(“Saliva” OR “salivas” OR “stimulated whole saliva” OR “salivary flow ” OR “saliva flow” OR “salivary flow rate” OR “resting whole saliva” OR “saliva flow rate” OR “unstimulated flow rates” OR “unstimulated salivary flow” OR “salivary” OR “salivary glands” OR “gland, salivary” OR “glands, salivary” OR “salivary gland” OR “saliva stress markers ” OR “Ph” OR “hyposalivation”)  #2: TS=(“Denture, Complete” OR “Complete Denture” OR “Complete Dentures” OR “Dentures, Complete” OR “Denture, Complete, Lower” OR “Denture, Complete, Upper” OR “denture wearing patients” OR “denture wearers”) |
| Virtual Health Library by Latin American and Caribbean Health Sciences Literature  (n=85) | #1: "Saliva" OR "salivas" OR "stimulated whole saliva" OR "salivary flow " OR "saliva flow" OR "salivary flow rate" OR  "resting whole saliva" OR  "saliva flow rate" OR "unstimulated flow rates" OR "unstimulated salivary flow" OR "salivary" OR "salivary glands" OR "gland, salivary" OR "glands, salivary" OR "salivary gland" OR "saliva stress markers " OR "Ph" OR "hyposalivation" OR mh:("Saliva" OR "Salivary Glands") AND instance:"regional"  #2: "Denture, Complete" OR "Complete Denture" OR "Complete Dentures" OR "Dentures, Complete" OR "Denture, Complete, Lower" OR "Denture, Complete, Upper" OR "denture wearing patients" OR "denture wearers" OR mh:("Denture, Complete" OR "Denture, Complete, Lower" OR "Denture, Complete, Upper") AND instance:"regional" AND instance:"regional" |
| Embase  (n=231) | #1: ‘Saliva’/exp OR ‘salivas’ OR ‘salivary flow’ OR ‘salivation’/exp OR ‘salivary flow rate’/exp OR  ‘resting whole saliva’ OR  ‘saliva flow rate’/exp OR ‘unstimulated flow rates’ OR ‘unstimulated salivary flow rate’/exp OR ‘salivary’ OR ‘salivary glands’/exp OR ‘gland, salivary’ OR ‘glands, salivary’ OR ‘salivary glands’/exp OR ‘saliva stress markers’ OR ‘Ph’/exp OR ‘hyposalivation’/exp  #2: ‘Denture, Complete’/exp OR ‘Complete Denture’/exp OR ‘Complete Dentures’ OR ‘Dentures, Complete’ OR ‘Denture, Complete, Lower’ OR ‘Denture, Complete, Upper’ OR ‘denture wearing patients’ OR ‘denture wearers’ |
| Scielo  (n=12) | 1#: Saliva OR salivas OR (stimulated whole saliva) OR (salivary flow)  OR (saliva flow) OR (salivary flow rate) OR  (resting whole saliva) OR  (saliva flow rate) OR (unstimulated flow rates) OR (unstimulated salivary flow) OR salivary OR (salivary glands) OR (gland, salivary) OR (glands, salivary) OR (salivary gland) OR (saliva stress markers)  OR Ph OR hyposalivation  2#: (Denture, Complete) OR (Complete Denture) OR (Complete Dentures) OR (Dentures, Complete) OR (Denture, Complete, Lower) OR (Denture, Complete, Upper) OR (denture wearing patients) OR (denture wearers) |
| Open Grey  (n=0) | 1#: “Saliva” OR “salivas” OR “stimulated whole saliva” OR “salivary flow ” OR “saliva flow” OR “salivary flow rate” OR  “resting whole saliva” OR  “saliva flow rate” OR “unstimulated flow rates” OR “unstimulated salivary flow” OR “salivary” OR “salivary glands” OR “gland, salivary” OR “glands, salivary” OR “salivary gland” OR “saliva stress markers ” OR “Ph” OR “hyposalivation”  2#: “Denture, Complete” OR “Complete Denture” OR “Complete Dentures” OR “Dentures, Complete” OR “Denture, Complete, Lower” OR “Denture, Complete, Upper” OR “denture wearing patients” OR “denture wearers” |
| Google Scholar  (n=100) | 1#: Saliva OR Salivary Flow OR Hyposalivation AND Denture, Complete OR Denture Wearers |

**Table S2**: Salivary biomarkers reported in clinical studies, with mean and standard deviation at different time points. Follow-up is shown in hours (h), days, weeks (w), and months (m) after intervention. “NA” indicates data not available.

| **Authors**  **years** | **Patients Characteristics** | | | | | | **Biomarker** | **Unit** | **Follow-Up** | | | | |
| --- | --- | --- | --- | --- | --- | --- | --- | --- | --- | --- | --- | --- | --- |
|  | **Study Design** | **Sample Size (n)** | **Sex** | **Age (Range/ Mean)** | **Previous Experience** | **Systemic Condition** |  |  | **Before** | **Hrs After** | **Days After** | **Week After** | **Months After** |
| Bhat *et al.,* 2014 | N-RCT | 20 | M (12)  F (8) | 52.55 ± 9.90 | NR | NR | **C- Reactive Protein (CRP)** | pg/mL | 475.42 ± 913.53 | NA | NA | NA | 657.98 ± 1326.69 (1m)**↑** |
|  |  |  |  |  |  |  | **Uric acid (UC)** | mg/dL | 2.79 ± 1.43 | NA | NA |  | 2.26 ± 1.17 (1m) **↓** |
| Reddy *et al.,* 2024 | N-RCT | 25 | M (11)  F (14) | 53 ± 2 | No | Healthy | **Salivary Alpha-Amylase (SAA**) | nm/ml | 8.26 ± 1.32 | 5.94 ± 1.35 **↓** | NA | 3.44 ± 1.25 (1w) **↓** | NA |
|  |  |  |  |  |  |  | **Nitric oxide (NO)** | nm/ml | 0.58 ± 0.29 | 6.56 ± 1.61**↑** | NA | 22.92 ± 5.79 (1w) **↑** | NA |
|  |  |  |  |  |  |  | **Thiobarbituric acid reactive substances (TBARS)** | nm/ml | 0.20 ± 0.05 | 3.27 ± 1.64 **↑** | NA | 14.33 ± 4.72 (1w) **↑** | NA |
| Breseghelo *et al.,* 2016 | N-RCT | 19 | M (5)  F (14) | 64.40 ± 8.30 | NR | Healthy | **Nitric oxide (NO)** | μM | 39.51 ± 11.79 | NA | 25.89 ± 5.54 (1st) **↓** 30.42 ± 6.76 (2nd) **↓** | NA | 44.81 ± 10.78  (10-12 m) ↑ |
| Bošković *et al.,* 2024 | N-RCT | 40 | NR | 28 - 78 (MD = 56) | NR | Healthy | **Malondialdehyde (MDA)** | nmol/mg | 10.19 ± 2.49 | 11.38 ± 5.92 **↑** | 11.73 ± 2.30 (1day) ↑ | 15.63 ± 2.34 (1w) ↑ | 11.19 ± 1.70 (1m) **↑** |
|  |  |  |  |  |  |  | **Advanced oxidation protein products (AOPP)** | µmol/mg | 36.42 ± 7.20 | 35.74 ± 5.84 **↓** | 49.15 ± 7.65 (1day)↑ | 59.00 ± 6.31 (1w)↑ | 42.84 ± 5.44 (1m)↑ |
|  |  |  |  |  |  |  | **NF-κB** | log fluorescence | 12.16 ± 1.85 | 16.80 ± 2.72 ↑ | 18.36 ± 3.09 (1day)↑ | 20.41 ± 3.84 (1w)↑ | 17.60 ± 2.48 (1m)↑ |
|  |  |  |  |  |  |  | **Catalase (CAT)** | U/g protein | 37.02 ± 6.30 | 28.60 ± 3.57**↓** | 24.46 ± 2.69 (1day)**↓** | 22.57 ± 4.37 (1w)**↓** | 29.80 ± 3.50 (1m)**↓** |
| Kostic *et al.,* 2019 | N-RCT | 6 | M (6) | 70 - 75 (MD = 72.33) | Yes | Healthy | **Tumor necrosis factor (TNF)-α** | pg/mL | 4.82 ± 2.05 |  | 4.79 ±3.34 (1 day)**↓** 6.31 ± 9.52 (3 day)↑ | 7.73 ± 4.006 (1w)↑ | 9.16±4.84 (1m)↑ |
|  |  |  |  |  |  |  | **Myeloperoxidase (MPO)** | U/μg | 3.45 ±1.89 | NA | 3.49 ± 2.38 (1day)**↑** 3.401 ± 1.59 (3 day)**↓** | 5.24 ±5.468 (1w)↑ | 3.27 ± 0.87 (1m)**↓** |
|  |  |  |  |  |  |  | **Immunoglobulin E (IgE)** | U/ml | 0.30 ± 0.00 | NA | 0.30 ± 0.00 (1 day) ↔ 0.30 ± 0.00 (3 day)↔ | 0.30 ± 0.00 (1w)↔ | 0.30 ± 0.00 (1m)↔ |
| El Naggar *et al.,* 2018 | N-RCT | 7 | NR | 45 - 60 | No | Healthy | **Salivary IgA** | mg/dL | 90 ± 6.24 | 92 ± 3.02↑ | 113 ± 7.47 (3 day)↑ | 132 ± 8.97 (1w)↑ | NA |
| Goiato *et al.,* 2019 | N-RCT | 41 | M (15)  F (26) | 73.70 ± 9.30 | Yes | NR | Salivary cortisol (SC) | NR | 16.84 ± 14.19 | NA | NA | NA | 9.35 ± 8.30 (1m)**↓** |
| Živković *et al.,* 2020 | N-RCT | 30 | M (13)  F (17) | 65 | No | Healthy | Salivary Alpha-Amylase (SAA) | U/ml | 264.0 ± 49.9 | 290.0 ± 50.9 ↑ | NA | NA | 318.0 ± 44.3 (2m)↑ |

Biomarker categories: inflammatory biomarkers (CRP, TNF-α, NF-κB, MPO, IgE, and salivary IgA), oxidative stress biomarkers (UA, NO, TBARS, MDA, AOPP, and CAT), and stress-related biomarkers (SC and SAA). Direction of change relative to baseline at each reported follow-up period: increase (↑), decrease (↓), and no change (↔). Stimulated and unstimulated salivary flow rates, expressed as mL/min.

**Figure S1**- Forest plots showing differences in unstimulated and stimulated salivary flow before and after complete denture (CD) insertion. (A) Immediately after CD insertion in individuals with and without CD experience. (B) 2-3 months after CD insertion, including both stimulated and unstimulated saliva, and participants with and without CD experience

**Figure S2.** Forest plots of the mean difference in unstimulated and stimulated salivary pH before and after complete denture (CD) insertion. **(A)** Immediately after CD insertion in individuals without previous experience. **(B)** Immediately after CD insertion in individuals with and without previous experience. **(C)** 2–3 months after CD insertion in individuals without previous experience. **(D)** 2–3 months after CD insertion in individuals with and without previous experience.
